# Phosphorylation of cyclin O, a novel cyclin family protein containing a cyclin-like domain, is involved in the activation of cyclin-dependent kinase 2

**DOI:** 10.3892/ol.2014.2530

**Published:** 2014-09-12

**Authors:** DO HYUNG KIM, JONG-HWA PARK, BORA LEE, KYOUNG OK JANG, IN SIK CHUNG, YE SUN HAN

**Affiliations:** 1 Department of Genetic Engineering and Graduate School of Biotechnology, Kyung Hee University, Giheung-gu, Yongin-si, Gyeonggi-do 446-701, Republic of Korea; 2 Department of Advanced Technology Fusion, Konkuk University, Hwayang-dong, Gwangjin-gu, Seoul 143-701, Republic of Korea

**Keywords:** cyclin-dependent kinase 2, cyclin O, phosphorylation, kinase activity

## Abstract

Cell cycles, ordered series of events modulating cell growth and division, are tightly regulated by complexes containing cyclin-dependent kinases (CDKs) and cyclins. Cyclin O is a novel cyclin family protein which interacts with CDK2. However, the molecular effects of cyclin O on the activity of CDK2 have not been fully evaluated. In this study, an interaction between cyclin O and CDK2 was identified by co-immunoprecipitation and the effect of cyclin O on the kinase activity of CDK2 was investigated using cyclin O point mutants. Co-immunoprecipitation was achieved using using HEK293 human embryonic kidney cells which were transiently transfected with vectors expressing cyclin O and CDK2, which revealed that cyclin O interacted with CDK2, particularly with the active form of endogenous CDK2. Cyclin O was expressed as several different bands with molecular weights between 45 and 50 kDa, possibly due to different post-translational modifications. When co-expressed with CDK2, cyclin O appeared as a band with a molecular weight of 50 kDa. Treatment with calf intestinal phosphatase reduced the intensity of the uppermost band. Mass spectroscopic analysis of cyclin O co-expressed with CDK2 revealed that the 81st serine residue of cyclin O was phosphorylated. The *in vitro* kinase activity of CDK2 phosphorylating histone H1 was markedly increased in the cells overexpressing cyclin O. This activity was reduced in cells overexpressing cyclin O, in which the 81st serine had been replaced with alanine (S81A). These results suggest that cyclin O is a novel cyclin family protein that regulates CDK2 kinase activity, which is mediated by the phosphorylation of the 81st serine residue of cyclin O.

## Introduction

The cell cycle, a series of events regulating cell division and duplication, is a ubiquitous, complex process involved in the growth and proliferation of cells, organism development, regulation of DNA damage repair and diseases such as cancer. The cell cycle involves numerous regulatory proteins that direct the cell through specific events culminating in mitosis and the production of two daughter cells. The cell cycle consists of four distinct phases: G1 phase, S phase (synthesis), G2 phase (interphase) and M phase (mitosis) (1,2).

The cell cycle is tightly regulated by complexes containing cyclin-dependent kinases (CDKs) and cyclins. The CDKs that belong to the Ser/Thr protein kinase family are typical cell cycle regulatory proteins. The catalytic activity of CDKs, phosphorylating proteins involved in diverse cell cycle processes, is tightly regulated by interactions with cyclin family proteins. Several CDKs have been identified and four have been shown to be active during the cell cycle: During G1 phase, CDK4, CDK6 and CDK2; during S phase, CDK2; during G2 and M phase, CDK1 (1). The levels of CDKs remain stable during the cell cycle. However, CDK activity is differentially regulated by cyclins, a family of proteins that control the progression of cells through the cell cycle by activating CDKs (3). Different cyclins are required at different phases of the cell cycle and the levels of cyclins are modulated during the cell cycle. Cyclin D synthesis is initiated during the G0 to G1 transition. Three subtypes of cyclin D (cyclin D1, cyclin D2 and cyclin D3) bind to CDK4 and CDK6. These CDK-cyclin D complexes are essential for cell entry into G1 (4). Cyclin E associating with CDK2 regulates progression between G1 and S phase (5,6). Cyclin A interacts with CDK2 and is required during S phase (7–9). Cyclin A also interacts with CDK1 in late G2 and early M phase and promotes entry into M phase. Mitosis is further regulated by the CDK1-cyclin B complex, which is involved in the early stages of mitosis including chromosome condensation, nuclear envelope breakdown, and spindle pole assembly (10). Cyclin H combines with CDK7 and the CDK7-cyclin H complex acts as a CDK-activating kinase (11,12).

Cyclin O is a novel cyclin family protein containing a cyclin-like domain, which is conserved in the cyclin family of proteins. Although cyclin O has been demonstrated to interact with CDK2 and is suggested to be required for the intrinsic apoptosis signaling pathway in lymphoid cells (13), the underlying molecular mechanism has not been fully evaluated. In the present study, the interaction between cyclin O and CDK2 was examined. The effect of cyclin O on the kinase activity of CDK2 was further investigated.

## Materials and methods

### Cell culture

HEK 293 human embryonic kidney cells obtained from the Korean Cell Line Bank (Seoul, Korea) were maintained at 37°C in a humidified atmosphere of 5% CO_2_ in Dulbecco’s modified Eagle’s medium supplemented with 10% heat-inactivated fetal bovine serum. The HEK 293 cells were seeded onto six-well plates at a density of 5×10^5^ cells per well or 60-cm^2^ culture dishes at a density of 2.5×10^6^ cells per dish, and incubated for 24 h prior to the experiments. The BL21 (DE3) *Escherichia coli* strain (EMD Chemicals, Inc., San Diego, CA, USA) served as a host for the cloning and expression of recombinant cyclin O deletion mutants. All cell culture medium and reagents were purchased from Hyclone™ (Thermo Fisher Scientific, Inc., Logan, UT, USA).

### Construction of expression vectors

Gene fragments corresponding to the coding regions of CDK2 and cyclin O (GenBank accession nos. NM001798 and NM021147, respectively) were amplified by polymerase chain reaction (PCR). The amplified DNA fragments were cloned into a T/A cloning vector, pGEM-T (Promega Corporation, Madison, WI, USA). The identity of the PCR DNA fragments was confirmed by restriction enzyme mapping and DNA sequence analysis. The cyclin O fragment was inserted between the *Eco*RI and *Sal*I sites of pCMV Tag3A (Stratagene California, La Jolla, CA, USA) to generate pCMV Tag3A/CyO. The CDK2 fragment was inserted between the *Bam*HI and *Sal*I sites of pCMV Tag2C (Stratagene California) to generate pCMV Tag2C/CDK2.

pCMV Tag3A/CyO S81A, a plasmid containing the *cyclin O* gene with a point mutation whereby the 81st serine residue is replaced with alanine, was generated by site-directed mutagenesis using the QuikChange site-directed mutagenesis kit (Stratagene California). The PCR primers used in mutagenesis were as follows: Sense, 5′-GCG GCG CGG GGT GGTGCC CCC CTG CCC GGC CCG-3′; and anti-sense, 5′-CGG GCC GGG CAG GGG GGC ACC CCA CGC CGC-3′. All constructs were further verified with restriction enzyme mapping and DNA sequence analyses.

### Transient expression of CDK2 and cyclin O

The expression vectors were transiently transfected into 80–90% confluent HEK 293 cells in six-well plates or 60-cm^2^ dishes using Lipofectamine^TM^ 2000 reagent (Invitrogen Life Technologies, Carlsbad, CA, USA) according to the manufacturer’s instructions. After 24 h incubation, the cells were lysed in lysis buffer [containing 50 mm Tris-HCl (pH 8.0), 100 mm NaCl, 5 mm EDTA, 1 mm NaF, 1 mm Na_3_VO_4_, 1% Nonidet P-40, 10 μg/ml PMSF, protease and phosphatase inhibitor cocktail (Sigma-Aldrich, St. Louis, MO, USA)] for 1 h at 4°C with occasional vortexing. Following centrifugation at 20,000 × g for 20 min, the cell supernatants were collected and used in western blot analysis and immunoprecipitation experiments.

### Co-immunoprecipitation

Total cell lysates were collected from the HEK 293 cells transfected with different sets of expression vectors, and then pre-cleared with 30 μl protein A/G-Sepharose beads (Santa Cruz Biotechnology, Inc., Santa Cruz, CA, USA) to remove nonspecific proteins. Following 1 h of incubation, centrifugation was conducted at 600 × g for 5 min, to separate the cell lysates from the beads. The pre-cleared supernatants were then incubated for 3 h with 2 μg mouse monoclonal anti-human c-myc or synthetic flag antibodies (sc-3777551 and sc-807, respectively; Santa Cruz Biotechnology, Inc.), and then incubated for 12 h with 30 μl protein A/G-Sepharose beads at 4°C under gentle rotation. The protein-bead complexes were precipitated by centrifugation at 600 × g for 5 min, washed five times with washing buffer [1:1 mixture of lysis buffer and phosphate-buffered saline (PBS)] and mixed with 2× sodium dodecyl sulfate-polyacrylamide gel electrophoresis (SDS-PAGE) loading buffer. Subsequent to boiling for 5 min, the immunoprecipitated samples were resolved on SDS polyacrylamide gel and subjected to western blot analysis.

### Identification of Ser^81^ phosphorylation by mass spectrometry

Total cell lysates were collected from the HEK 293 cells co-transfected with vectors expressing c-myc-tagged cyclin O and flag-tagged CDK2. The cell lysates (1 mg) pre-cleared with 30 μl protein A/G-Sepharose beads were immunoprecipitated with anti-c-myc as described above. The immunoprecipitated samples were resolved on SDS polyacrylamide gel and visualized by silver staining. The region with cyclin O was excised from the SDS polyacrylamide gel. The proteins were reduced, alkylated and then digested with 12.5 ng/μl sequencing grade modified trypsin (Promega Corporation), which was followed by digestion with endoproteinase Glu-C (Roche Diagnostics GmbH, Mannheim, Germany). The digested peptides were extracted three times with 5% formic acid in 50% acetonitrile solution at room temperature for 20 min, and then desalted using C18 ZipTips (Millipore, Billerica MA, USA) prior to mass spectrometry (MS) analysis. The proteolytic peptides were loaded onto a fused silica microcapillary column (12 cm × 75 μm) packed with the C18 reversed-phase resin (5 μm, 200 Å). Liquid chromatography separation was performed for 60 min under a 3–40% solvent B (0.1% formic acid in 100% acetonitrile) linear gradient, with a flow rate of 250 ml/min. The column was directly connected to an LTQ linear ion-trap mass spectrometer (ThermoFinnigan, San Jose, CA, USA) equipped with a nano-electrospray ion source. The electrospray voltage was set at 2.05 kV, and the threshold for switching between MS and MS/MS was 500. The normalized collision energy for MS/MS was 35% of the main radio frequency amplitude and the duration of activation was 30 msec. All spectra were captured in data-dependent scan mode. Each full MS scan was followed by five MS/MS scans corresponding to the most intense up to the fifth most intense peaks of the full MS scan. The repeat count of the peak for dynamic exclusion was 1 and the repeat duration was 30 sec. The dynamic exclusion duration was 180 sec and the exclusion mass width was ±1.5 Da. The dynamic exclusion list size was 50. The collected MS/MS spectra were searched using the Sequest program (Thermo Fisher Scientific, Inc.) with oxidation on Met (+16 Da), carboxyamidomethylation on Cys (+57 Da) and phosphorylation on Ser, Thr and Tyr (+80 Da) as selected criteria for variable modifications.

### In vitro CDK2 kinase activity assay

The cell lysates (1 mg) pre-cleared with 30 μl protein A/G-Sepharose beads were incubated with 1 μg rabbit polyclonal anti-human CDK2 (Santa Cruz Biotechnology, Inc.) or goat polyclonal anti-rabbit IgG, which was followed by 12 h of incubation with 30 μl protein A/G-Sepharose beads at 4°C under gentle rotation. The protein-bead complexes were precipitated by centrifugation at 600 × g for 5 min, washed three times with washing buffer (1:1 mixture of lysis buffer and PBS) and twice with kinase assay buffer [containing 40 mm Tris-HCl (pH 7.5) and 10 mm MgCl_2_]. The final protein-bead complexes were resuspended in 20 μl kinase assay buffer and divided into two tubes. One tube was used to determine the presence of CDK2 and the other was used to examine the kinase activity that phosphorylated histone H1 (HH1; New England Biolabs, Ipswich, MA, USA). In the kinase activity assay, protein-bead complexes were added to 1 μg HH1, 10 μM ATP, 0.5 mm dithiothreitol, 0.5 mm EGTA, 50 mm β-glycerophosphate, 1 mm NaF, 0.1 mm sodium orthovanadate and 10 μCi [γ-^32^P] ATP (Amersham Biosciences, Chalfont St. Giles, UK). Subsequent to incubating for 1 h at room temperature, the reactions were terminated by the addition of 5× SDS-PAGE loading buffer. The reaction mixtures were boiled for 2 min and separated from the Sepharose beads by centrifugation at 16,000 × g for 10 min. The reaction mixtures were resolved by SDS-PAGE (15% SDS polyacrylamide gel) and then dried. [γ-^32^P]-incorporated HH1 was visualized by autoradiography (Kodak XAR film; Kodak, Rochester, NY, USA) and quantified using TINA densitometry software version 2.09c (Raytest Isotopenmessgeraete GmbH, Straubenhardt, Germany).

### Western blot analysis

The cell lysates were resolved on 10 or 12% SDS polyacrylamide gels and transferred to polyvinylidenedifluoride membranes (PALL Life Science, Pensacola, FL, USA). The membranes were blocked for 1 h at room temperature with 3% non-fat dried milk in Tris-buffered saline with 0.1% Tween-20 (TBS-T), and then incubated overnight at 4°C with primary antibody solutions [mouse monoclonal anti-human c-myc, mouse monoclonal synthetic flag (Sigma-Aldrich) or anti-human CDK2 (Santa Cruz Biotechnology, Inc.) resuspended in TBS-T containing 3% non-fat dry milk]. The membranes were washed three times with TBS-T and incubated for 2 h with secondary antibody solutions [horseradish peroxidase-conjugated goat polyclonal anti-mouse or anti-rabbit IgG (Santa Cruz Biotechnology, Inc.) resuspended in TBS-T containing 3% non-fat dry milk]. Following washing with TBS-T, the protein bands were detected using SuperSignal West Pico chemiluminescence substrate (Pierce, Rockford, IL, USA).

## Results

### Cyclin O interacts with CDK2, particularly with the active form

**Figure 1:**
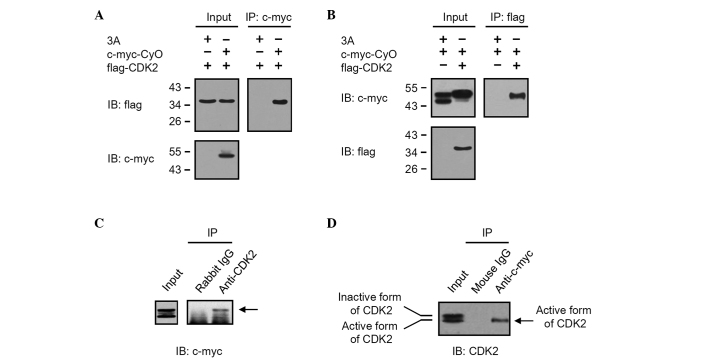
Cyclin O interacted with CDK2. (A) HEK 293 human embryonic kidney cells were transfected with different sets of vectors expressing c-myc-CyO and flag-CDK2. Following co-immunoprecipitation using anti-c-myc, flag-CDK2 interaction with c-myc-CyO was determined by western blot analysis using anti-flag. ‘3A’ signifies an empty vector, pCMV Tag3A. (B) Co-immunoprecipitation was also performed with anti-flag. The presence of c-myc-CyO in immunoprecipitates was determined by western blot analysis using anti-c-myc. (C) HEK 293 cell extracts transiently transfected with vector expressing c-myc-CyO were immunoprecipitated with rabbit IgG or anti-CDK2. The presence of c-myc-CyO in immunoprecipitates was determined by western blot analysis. The arrow indicates c-myc-CyO co-immunoprecipitated by anti-CDK2. (D) HEK 293 cell extracts were also immunoprecipitated with mouse IgG or anti-c-myc. The presence of endogenous CDK2 in the immunoprecipitates was determined by western blot analysis. CyO, cyclin O; CDK2, cyclin-dependent kinase 2; IP, immunoprecipitation; IB, immunoblotting.

The interaction between cyclin O and CDK2 was determined by co-immunoprecipitation using transiently transfected HEK 293 cells. Different sets of vectors (pCMV Tag3A/CyO, pCMV Tag2C/CDK2) expressing c-myc-tagged cyclin O (c-myc-CyO) and flag-tagged CDK2 (flag-CDK2), as described in Fig. 1A and B, were transiently transfected into HEK 293 cells. After 24 h of incubation, total cell lysates were harvested and immunoprecipitated with anti-c-myc or anti-flag. Western blot analysis using the anti-flag signal of samples immunoprecipitated with anti-c-myc revealed that flag-CDK2 was co-immunoprecipitated by interacting with c-myc-CyO (Fig. 1A). In addition, western blot analysis using the anti-c-myc signal of samples immunoprecipitated with anti-flag also demonstrated that c-myc-CyO was co-immunoprecipitated by interacting with flag-CDK2 (Fig. 1B). The interaction between cyclin O and CDK2 was further determined by co-immunoprecipitation of endogenous CDK2 from HEK 293 cell extracts transfected with pCMV Tag3A/CyO (Fig. 1C and D). The total cell lysates obtained from HEK 293 cells transiently transfected with pCMV Tag3A/CyO were immunoprecipitated with rabbit IgG or anti-CDK2. Western blot analysis using anti-c-myc revealed that c-myc-CyO was co-immunoprecipitated by anti-CDK2, but not rabbit IgG (Fig. 1C). When the total cell lysates were immunoprecipitated with mouse IgG or anti-c-myc, the active form of endogenous CDK2 was also co-immunoprecipitated by anti-c-myc (Fig. 1D).

### Cyclin O is phosphorylated by CDK2

**Figure 2:**
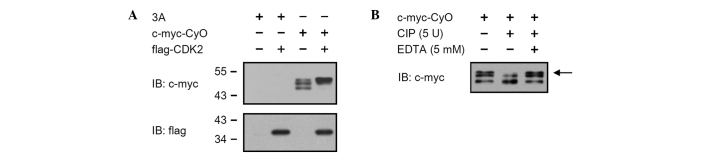
Phosphorylation of c-myc-CyO was enhanced by the co-expression of CDK2. (A) HEK 293 human embryonic kidney cells were transiently transfected with vectors expressing c-myc-CyO and/or flag-CDK2. The expression levels of c-myc-CyO and flag-CDK2 were determined by western blot analysis. (B) HEK 293 cell lysates expressing c-myc-CyO were incubated with 5 U CIP in the presence or absence of 5 mm EDTA. The reaction mixtures were analyzed by western blotting using anti-c-myc. The arrow indicates the uppermost band that disappeared with CIP treatment, although this effect was reversed when EDTA was added. CyO, cyclin O; CDK 2 cyclin-dependent kinase 2; CIP, calf intestinal phosphatase; IB, immunoblotting.

HEK 293 cells were transiently transfected with pCMV Tag3A/CyO in the presence or absence of pCMV Tag2C/CDK2, and the expression levels of c-myc-CyO were determined by western blot analysis. c-myc tagged cyclin O was expressed as three bands with molecular weights between 45 and 50 kDa. When co-expressed with flag-CDK2, c-myc-CyO was expressed as a single band with a molecular weight of 50 kDa (Fig. 2A). To determine whether the cyclin O was phosphorylated, the cell lysates of the HEK 293 cells transiently transfected with pCMV Tag3A/CyO were treated with 5 units of calf intestinal phosphatase (CIP) for 1 h at 30°C. The uppermost band of c-myc-CyO disappeared in CIP-treated cell lysates (Fig. 2B, lane 2), but not in cell lysates treated with CIP and EDTA (Fig. 2B, lane 3).

### CKD2 phosphorylates the 81st serine residue of cyclin O

**Figure 3:**
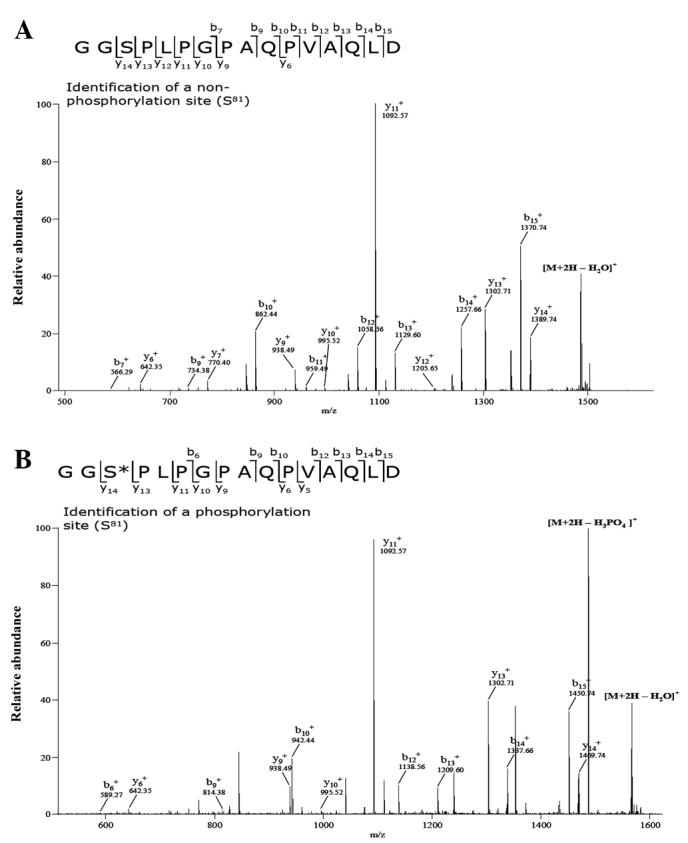
Cyclin O 81st serine (S81) residue was phosphorylated following co-expression of cyclin O and CDK2. HEK 293 human embryonic kidney cells were transfected with vectors expressing c-myc-CyO and flag-CDK2. Phosphorylated and non-phosphorylated cyclin O were excised from a sodium dodecyl sulfate-polyacrylamide gel and analyzed using an LTQ linear ion-trap mass spectrometer. The collected MS/MS spectra were searched using the Sequest program. (A) and (B) indicate the identities of the non-phosphorylated and phosphorylated site of Ser81, respectively. CDK2, cyclin-dependent kinase 2; CyO, cyclin O; MS, mass spectroscopy.

**Figure 4:**
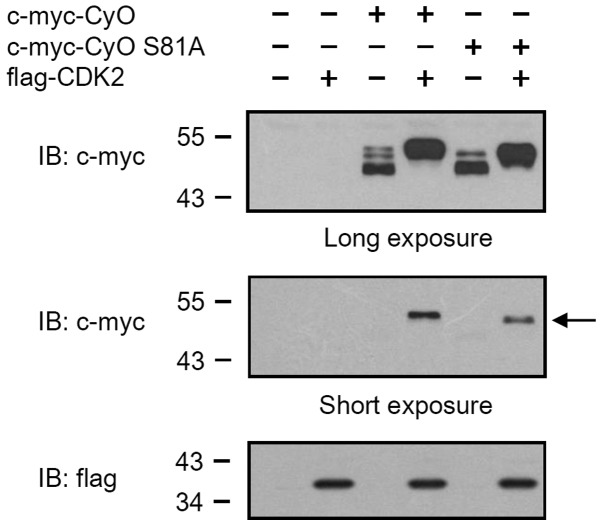
The uppermost band of cyclin O was not detected in cyclin O S81A. HEK 293 human embryonic kidney cells were transiently transfected with vectors expressing c-myc-CyO and c-myc-CyO S81A in the presence or absence of vector expressing flag-CDK2. The expression levels and phosphorylation of c-myc-CyO and c-myc-CyO S81A were determined by western blot analysis. The arrow indicates the band of c-myc-CyO S81A co-expressed with flag-CDK2. CyO, cyclin O; CDK2, cyclin-dependent kinase 2; IB, immunoblotting.

To determine which amino acid residues are phosphorylated by CDK2, total cell lysates were collected from the HEK 293 cells co-transfected with vectors expressing c-myc-CyO and flag-CDK2. Subsequently, 1 mg cell lysate was pre-cleared with 30 μl protein A/G-Sepharose beads and immunoprecipitated with anti-c-myc. The immunoprecipitated samples were resolved on SDS polyacrylamide gel and visualized by silver staining (data not shown). The regions with the phosphorylated and non-phosphorylated cyclin O bands were excised from the SDS polyacrylamide gel. The proteins were reduced, alkylated and digested with trypsin. MS analysis of the digested peptides revealed that sizes of the peptides including the 81st serine residue of phosphorylated c-myc-CyO were greater (~80 Da) than those obtained from non-phosphorylated c-myc-CyO (Fig. 3A and B). This result indicates that the 81st serine residue of cyclin O was phosphorylated, which may be caused by CDK2. To further examine whether the phosphorylation of the 81st serine residue of cyclin O is caused by CDK2, a cyclin O gene with a point mutation (cyclin O S81A) was generated by replacing the 81st serine residue with an alanine, and was transiently expressed in HEK 293 cells. As shown in Fig. 4, the c-myc-tagged cyclin O S81A (c-myc-CyO S81A) was expressed as two bands (lane 5). When co-expressed with flag-tagged CDK2, c-myc-CyOS81A was expressed as a band with a molecular weight of 48 kDa (lane 6), which was smaller than the band that appeared when cyclin O was co-expressed with CDK2 (lane 4).

### Following phosphorylation of the 81st serine residue, cyclin O stimulates in vitro CDK2 kinase activity, which involves the phosphorylation of HH1

**Figure 5:**
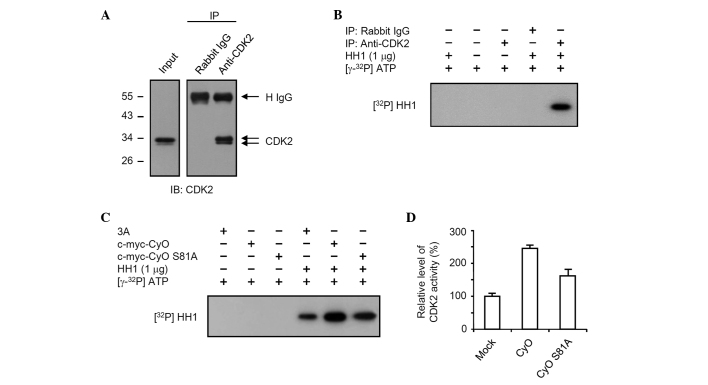
*In vitro* CDK2 kinase activity was increased by cyclin O. (A) HEK 293 cell lysates were immunoprecipitated with rabbit IgG or anti-CDK2. The presence of CDK2 in the immunoprecipitates was analyzed by western blotting. (B) The immunoprecipitates were incubated with 1 μg HH1 and 10 μCi [γ-^32^P] ATP. [γ-^32^P]-incorporated HH1 was visualized by autoradiography. (C) Immunoprecipitates following anti-CDK2 treatment were obtained from the HEK 293 cells expressing c-myc-CyO or c-myc-CyO S81A, and were incubated with 1 μg HH1 and 10 μCi [γ-^32^P] ATP. [γ-^32^P]-incorporated HH1 was visualized by autoradiography. (D) The CDK2 kinase activity of three independent immunoprecipitation experiments in (C) is presented as a bar diagram. Data are presented as the mean ± standard deviation. CDK2, cyclin-dependent kinase 2; H IgG, IgG heavy chain; HH1, histone H1; CyO, cyclin O; IP, immunoprecipitation.

To assess the *in vitro* kinase activity of CDK2, HEK 293 cell lysates were immunoprecipitated with anti-CDK2 or rabbit IgG as a control. CDK2 was immunoprecipitated by anti-CDK2, but not rabbit IgG (Fig. 5A). The immunoprecipitates were incubated with HH1 in the presence of [γ-^32^P] ATP. [γ-^32^P]-incorporated HH1 was detected in the anti-CDK2 immunoprecipitate reaction mixture (Fig. 5B, lane 5), but not in the rabbit IgG reaction mixture (Fig. 5B, lane 4). The cell lysates obtained from the HEK 293 cells transiently transfected with vectors expressing c-myc-CyO or c-myc-CyO S81A were immunoprecipitated with anti-CDK2, and a kinase activity assay was performed. The CDK2 kinase activities of the immunoprecipitates obtained from the HEK 293 cells that were transiently transfected with vectors expressing c-myc-CyO and c-myc-CyO S81A were increased by 2.5- and 1.6-fold, respectively, compared with those of the HEK 293 cells transiently transfected with the empty vector, pCMV Tag3A (Fig. 5C and D).

## Discussion

Cyclin O was originally identified as a cyclin-like uracil DNA glycosylase and was suggested to function as a putative base excision repair DNA glycosylase with the ability to excise uracil from U:A or U:G mismatched DNA duplexes (14). However, in our previous investigations, the recombinant cyclin O produced by *E. coli* and a mammalian expression system did not contain any uracil DNA glycosylase activity (data not shown). Hirst *et al* (15) identified murine cyclin O in murine oocytes, and proposed that cyclin O may be associated with oocyte development and maturation. Recently, cyclin O has been reported to interact with CDK2, a molecule belonging to the Ser/Thr protein kinase family that is involved in normal cell cycle progression (13). Cyclin O is required for the intrinsic apoptosis signaling pathway in lymphoid cells (13). However, the molecular mechanism of the interaction between cyclin O and CDK2, which is involved in normal cell cycle progression, has not been fully analyzed. In the present study, the physical interaction between cyclin O and CDK2, and the effect of cyclin O on the catalytic activity of CDK2, were investigated.

In co-immunoprecipitation experiments using transiently transfected HEK 293 cells, c-myc-tagged cyclin O interacted with flag-tagged CDK2 and endogenous CDK2 (Fig. 1). In HEK 293 cells, flag-tagged CDK2 was expressed as a band with a molecular weight of ~37 kDa. However, endogenous CDK2 was expressed as two bands. One band was a highly phosphorylated inactive form of CDK2 [CDK2 phosphorylated at the 14th threonine (T14), 15th tyrosine (Y15) and 160th threonine (T160)] and the other was a simply phosphorylated active form of CDK2 (CDK2 phosphorylated at the 160th threonine) (16). Cyclin O was shown to interact with the active form of endogenous CDK2 (Fig. 1D). These results demonstrate that cyclin O interacts with CDK2, particularly with the active form.

Human and mouse cyclin O have been reported to be expressed as three and two bands, respectively (15). In the present study, c-myc-CyO in HEK 293 cells was expressed as three bands with molecular weights between 45 and 50 kDa. The overexpression of cyclin O has been suggested to induce caspase-dependent apoptosis in cell lines of non-lymphoid origin (15). However, in the present study, caspase-dependent apoptosis was not observed in HEK 293 cells transiently overexpressing cyclin O (data not shown). When co-expressed with flag-CDK2, c-myc-CyO appeared as a band with a molecular weight of 50 kDa (Figs. 1 and 2). The finding that the increase in the signal from the uppermost band (with a molecular weight of 50 kDa) is caused by the co-expression of cyclin O with CDK2, indicates that cyclin O is present as differently modified forms, possibly due to different post-translational modifications. This is most likely due to phosphorylation regulation by CDK2. The uppermost band of c-myc-CyO disappeared in CIP-treated cell lysates (Fig. 2B). This indicates that the uppermost cyclin O band was in a phosphorylated form. This also suggests that the interaction between CDK2 and cyclin O may induce the phosphorylation of cyclin O.

In the MS analysis performed in the present study to identify the amino acid residues phosphorylated by CDK2, the 81st serine residue of cyclin O was shown to be phosphorylated. In addition, the uppermost 50 kDa cyclin O band was not observed in the cyclin O S81A mutant. These results indicate that the uppermost band of cyclin O may be caused by the phosphorylation of the 81st serine residue.

The CDK2 kinase activity of the immunoprecipitate obtained from the HEK 293 cells transiently transfected with a vector expressing c-myc-CyO was markedly increased when compared with that of the HEK 293 cells transiently transfected with the empty vector, pCMV Tag3A. The CDK2 kinase activity of cells overexpressing c-myc-CyO S81A was less than that of cells overexpressing native cyclin O. These results indicate that the CDK2 kinase activity was increased by cyclin O, which may be mediated by the phosphorylation of the 81st serine residue on cyclin O.

The results of our previous investigations also suggested that another post-translational modification of cyclin O is induced by CDK2. To determine which amino acid residues are modified and affect the shifted-motility of cyclin O, the cyclin O deletion mutants D1, D2 and D3, which correspond to amino acids 1–115, 116–350 and 231–350, respectively, were produced by bacterial and mammalian expression systems. The cyclin O deletion mutants D2 and D3 were expressed as bands with molecular weights of 32 and 20 kDa, respectively, in bacterial and mammalian expression systems (data not shown). However, in HEK 293 cells, the D1 cyclin O deletion mutant was expressed as 2–3 bands with molecular weights of 24–26 kDa, which were larger than the band obtained in the bacterial expression system (22 kDa; data not shown). These results suggest that the D1 cyclin O deletion mutant, corresponding to 1–115 amino acid residues, contains additional modification residues to enlarge the molecular weight. N-terminal deletion domains (NDD) were also generated from pCMV Tag3A/CyO S81A and sub-cloned into the mammalian expression vector, pCMV Tag3A. Cyclin O S81A NDD mutants containing 5–530, 16–350 amino acids were expressed as two bands with different molecular weights. However, the cyclin O S81A NDD mutant containing 51–350 amino acids was expressed as one band. This indicates that additional modifications may occur at amino acid residues between 16 and 50. However, in a preliminary experiment to determine whether additional modifications affect the kinase activity of CDK2, the cyclin O NDD mutant containing 51–350 amino acids and the 81st serine residue did not reduce the *in vitro* kinase activity of CDK2 stimulated by native cyclin O (data not shown). This signifies that additional modifications of N-terminal 50 amino acid residues are not involved in the activation of CDK2. Although additional studies are required to further determine the molecular mechanism of cyclin O involvement in CDK2 activity, the results of the present study suggest that CDK2 kinase activity stimulated by cyclin O is mediated by the phosphorylation of the 81st serine residue of cyclin O.

In conclusion, co-immunoprecipitation using transiently transfected HEK 293 cells revealed that cyclin O interacted with CDK2, particularly with the active form of endogenous CDK2. Cyclin O was expressed as three sizes, but when cyclin O was co-expressed with CDK2, the intensity of the uppermost band was increased. The phosphorylation of the 81st serine residue of cyclin O by CDK2 was confirmed through MS and expression analysis using the cyclin O S81A mutant. The *in vitro* kinase activity of CDK2 phosphorylating HH1 was increased in the cells overexpressing cyclin O, which exhibited higher activity than that of the cells overexpressing cyclin O S81A. The results indicate that CDK2 kinase activity was stimulated by the interaction with cyclin O. Therefore, the phosphorylation of the 81st serine residue of cyclin O is involved in the regulatory mechanism of CDK2 kinase activity by cyclin O.
